# Subjective Outcome Evaluation of the Project P.A.T.H.S. in Different Cohorts of Students

**DOI:** 10.1100/2012/493957

**Published:** 2012-08-01

**Authors:** Daniel T. L. Shek, Cecilia M. S. Ma

**Affiliations:** ^1^Department of Applied Social Sciences, Faculty of Health and Social Sciences, The Hong Kong Polytechnic University, Room HJ407, Core H, Hunghom, Hong Kong; ^2^Public Policy Research Institute, The Hong Kong Polytechnic University, Hong Kong; ^3^Department of Social Work, East China Normal University, Shanghai 200062, China; ^4^Kiang Wu Nursing College of Macau, Macau, China; ^5^Division of Adolescent Medicine, Department of Pediatrics, Kentucky Children's Hospital, University of Kentucky College of Medicine, Lexington, KY 40506, USA

## Abstract

The project P.A.T.H.S. is an indigenously developed positive youth development program in Hong Kong. In the extension phase (2009/2010 school year), subjective outcome evaluation data were collected from 231 schools involving 89,068 participants after completion of the curricula-based Tier 1 Program. With schools as the units of analysis, results showed that participants generally had positive perceptions of the program content and implementers, with over four-fifth of the participants regarded the program as helpful to them. There were some significant grade differences in the subjective outcome evaluation findings, although the related effect size was not strong. Multiple regression analyses revealed that program content and program implementers predicted perceived effectiveness of the program. The present study suggests that irrespective of cohorts, students in the junior secondary years perceived the program to be beneficial to them.

## 1. Introduction

The increasing popularity of implementing effective adolescent prevention programs in recent decades has been a key initiative to tackle adolescent developmental problems [1–4]. Researchers [5–7] identified eight factors that are essentials for the implementation of adolescent prevention programs. These include fidelity (i.e., the extent to which the program is implemented as originally designed), dosage (i.e., the number of sessions offered during implementation), quality of delivery (i.e., the extent to which the program is delivered in an authentic manner), participant responsiveness (i.e., participants' involvement and satisfaction), program differentiation (i.e., the extent to which a program's theory and practices can be distinguished from other available programs), monitoring (i.e., documenting the nature and amount of services received by the service recipients), program reach (i.e., the proportion of the intended audience who participated in the intervention), and adaptation (i.e., the extent to which the program is different from the original designed during implementation).

Research findings showed that positive attitudes toward the program content and program implementers were associated with program outcomes [8–13]. However, little is known about the relative influence of these factors on program effectiveness as prior studies mainly focused on one component only [6, 14–16]. For example, Rohrbach et al. [13] noted the interrelationships of these factors and suggested to explore their relative influences on program effectiveness in the future evaluation research. Berkel et al. [17, page 24] highlighted that “program evaluations have rarely examined more than one dimension in a single study and thus have not untangled possible relations between them”. To fill this gap, the present study explored the relative influence of two program implementation factors on perceived program outcomes.

Prevention researchers noted the importance of providing culturally competent interventions for a given population [18–20]. However, as adolescent prevention programs were predominantly conducted in the Western countries, it is not clear whether the previous findings would vary by different subgroups of participants, such as adolescents in non-Western contexts. This question makes sense because assuming that the application of concepts and behaviors is universal to every individual in a population is debatable which may lead to problematic results [21]. Catalano et al. [22] argued that more effort is needed “to understand how well they can be implemented in real-world settings and what effects they are likely to have…and examine differences of effects on relevant subgroups (e.g., culture, gender, age, etc.)” (page S93). It appears that findings from non-Western cultural contexts would certainly expand the scope of program evaluation literature.

The Project “P.A.T.H.S. to Adulthood: A Jockey Club Youth Enhancement Scheme” is a large-scale positive youth development program designed for junior secondary school students (Secondary 1 to 3, i.e., Grades 7 to 9) in Hong Kong [23]. The word “P.A.T.H.S.” denotes Positive Adolescent Training through Holistic Social Programmes. It consists of two tiers of program. The Tier 1 Program targets all students joining the program in a particular form (i.e., universal prevention initiative). Through the use of structured curriculum, students learn competencies with reference to the 15 positive youth development construct [23]. The Tier 2 Program is specially designed for students with greater psychosocial needs in different psychosocial domains (i.e., selective prevention). After completion of the Tier 1 Program, program participants were required to complete a subjective outcome evaluation form (Form A).

Qualitative and quantitative data collected based on the original phase of the project generally suggested that participants (students and program implementers) perceived the program positively [24–35]. However, little is known whether the impact of program implementation factors on program effectiveness would be sustained in the extension phase. Also, it is not clear whether these relationships would vary by students' grade level. In particular, the relative influence of these factors on program outcomes is relatively unexplored. Against the aforementioned background, the purpose of the study was to examine the effectiveness of the Tier 1 Program of the Project P.A.T.H.S. and to test the relative influence of two aspects of program implementation, namely, perceptions of the program (content as well as implementation) and program implementers on perceived program effectiveness. It also attempted to investigate whether the predictive effects of these factors would differ across grade levels.

## 2. Methods

### 2.1. Participants and Procedures

A total of 231 schools with 89,068 students joined the Project P.A.T.H.S. in the extension phase of the Full Implementation Phase in the school year 2009/2010. (The initial phase of the project was started from the academic year 2005/2006 to 2008/2009.) A total of 577 aggregated data sets from the participating schools were collected across three grade levels (i.e., Secondary 1 level: 219 schools; Secondary 2 level, 185 schools; and Secondary 3 level, 173 schools). The mean number of students per school was 154.36 (ranged from 6 to 240 students), with an average of 4.50 classes per school (ranged from 1 to 12 classes). Among them, 32.24% of the respondent schools adopted the full program (i.e., 20-hour program involving 40 units) whereas 67.76% of the respondent schools adopted the core program (i.e., 10-hour program involving 20 units). The mean number of sessions used to implement the program was 28.54 (ranged from 2 to 48 sessions). While 47.31% of the participating schools incorporated the program into the formal curriculum (e.g., Liberal Studies, Life Education), 52.69% used other modes (e.g., classes and events that differed from normal class schedule) to implement the program. The mean number of social workers and teachers implementing the program per school was 1.71 (ranged from 0 to 7) and 5.11 (ranged from 0 to 27), respectively.

After completion of the Tier 1 Program, the participants were invited to respond to a Subjective Outcome Evaluation Form (Form A) developed by the first author [36]. The data collection was carried out at the last session of the program. On the day of data collection, the purpose of the evaluation was mentioned, and confidentiality of the data was repeatedly emphasized to all students. The students were asked to indicate their wish if they did not want to participate in the study (i.e., passive informed consent was obtained from the students). All participants responded to all scales in the evaluation form in a self-administration format. Adequate time was provided for the participants to complete the questionnaire.

### 2.2. Instruments

The Subjective Outcome Evaluation Form (Form A) was used. Broadly speaking, there are several parts in this evaluation form as follows:

- participants' perceptions of the program, such as program objectives, design, classroom atmosphere, interaction among the students, and the respondents' participation during class (10 items);
- participants' perceptions of the program implementers, such as the preparation of the instructor, professional attitude, involvement, and interaction with the students (10 items);
- participants' perceptions of the effectiveness of the program, such as promotion of different psychosocial competencies, resilience, and overall personal development (16 items);
- the extent to which the participants would recommend the program to other people with similar needs (1 item);
- the extent to which the participants would join similar programs in the future (1 item);
- overall satisfaction with the program (1 item);
- things that the participants learned from the program (open-ended question);
- things that the participants appreciated most (open-ended question);
- opinion about the instructor(s) (open-ended question);
- areas that require improvement (open-ended question).

For the quantitative data, the implementers collecting the data in each school were requested to input the data in an EXCEL file developed by the research team which would automatically compute the frequencies and percentages associated with the different ratings for an item. When the schools submitted the reports, they were also requested to submit the soft copy of the consolidated data sheets. In the reports prepared by the schools, the workers were also required to estimate the degree of adherence to the program manuals (i.e., the extent to which the program is implemented in accordance with the program manuals). To facilitate the program evaluation, the research team developed an evaluation manual with standardized instructions for collecting the subjective outcome evaluation data [36]. In addition, adequate training was provided to the implementers during the 20-hour training workshops on how to collect and analyze the data collected by Form A. After receiving the consolidated data by the funding body, the data were aggregated to reconstruct the overall profile based on the subjective outcome evaluation data by the research team.

### 2.3. Data Analyses

Percentage findings were examined using descriptive statistics. A composite measure of each domain (i.e., perceived qualities of program, perceived qualities of program implementers, and perceived program effectiveness) was created based on the total scores of each factor divided by the number of items in that domain. Pearson correlation analysis was used to examine if the program content and program implementers were related to the program effectiveness. One-way analysis of variance (ANOVA) was used to assess the differences in the mean of each factor across grade levels. Multiple regression analysis was performed to compare which factor would predict the program effectiveness. All analyses were performed by using the Statistical Package for Social Sciences Version 19.0.

## 3. Results

Quantitative findings based on the closed-ended questions are presented in this paper. Several observations can be highlighted from the findings. In the first place, roughly four-fifth of the participants generally had positive perceptions of the program (Table 1), including clear objectives of the curriculum (85.32%), well-planned teaching activities (83.59%), and adequate peer interaction among the students (82.90%). In addition, a high proportion of the students had positive evaluation of the instructors (Table 2). For example, 89.44% of the participants perceived that the program implementers were very involved; 89% of the participants agreed that implementers encouraged them to participate in the activities; 88.86% perceived that the implementers were ready to offer help when they are in needs.

As shown in Table 3, more than four-fifth of the respondents perceived that the program promoted their development, including the ability to distinguish between the good and the bad (86.04%), competence in making sensible and wise choices (85.15%), ability to resist harmful influences (85.04%), and overall development (85.33%). Interestingly, while roughly three-quarter (78.55%) of the participants would recommend the program to their friends who have similar needs, only 67.79% of them would join similar programs in the future. Finally, more than four-fifth (85.65%) of the participants indicated that they were satisfied with the program (Table 4). Regarding the degree of program adherence estimated by the program implementers, the mean level of adherence was 83.50%, with a range from 14.5% to 100%.

Results of reliability analysis showed that Form A was internally consistent (Table 5): 10 items related to the program content (*α* = .98), 10 items related to the program implementers (*α* = .99), 16 items related to the benefits (*α* = 1.00), and the overall 36 items measuring program effectiveness (*α* = .99). Results of correlation analysis showed that both program content (*r* = .84, *P* < .01) and program implementers (*r* = .76, *P* < .01) were strongly associated with program effectiveness. These positive relationships were consistent across all grade levels (Table 6).

To examine differences in the subjective outcome measures (i.e., program content, program implementers, and program effectiveness) across levels, a series of one-way ANOVAs were performed with different subjective outcome indicators as dependent variables and grade level (i.e., Secondary 1 to 3 levels) as an independent variable. Significant results were found for program content (*F*_(2,574)_ = 6.07, *P* < .01), program implementers (*F*_(2,574)_ = 8.62, *P* < .01), program effectiveness (*F*_(2,574)_ = 11.51, *P* < .01), and the total scale (*F*_(2,574)_ = 9.85, *P* < .01) (Table 5).

Post hoc analysis using the Bonferroni adjustment (*P* = .02) revealed that significant differences were found between Secondary 1 (*M* = 4.37) and Secondary 2 (*M* = 4.26) students toward their perceptions on program content (*P* < .01) and their perceptions on program implementers (Secondary 1: *M* = 4.68, Secondary 2: *M* = 4.55, *P* < .01). Significant grade differences were also shown when comparing students' perceptions toward the program effectiveness (Secondary 1: *M* = 3.50, Secondary 2: *M* = 3.37; Secondary 3: *M* = 3.41, *P* < .01). Similar results were revealed in the overall program effectiveness (Secondary 1: *M* = 4.07, Secondary 2: *M* = 3.95, *P* < .01; Secondary 3: *M* = 4.00, *P* < .05). It is noteworthy that the previous differences were not significant between Secondary 2 and 3 classes (*P* > .05). Overall speaking, junior students perceived the program more effective than their senior counterparts. However, it is noteworthy that the effect size of grade differences was not strong.

Table 7 presents multiple regression analysis results. Program content was positively associated with perceived program effectiveness (*P* < .01). On the other hand, program implementer was not associated with program effectiveness (*P* > .05). However, the result based on the Secondary 2 students showed that perception toward the program implementer was negatively associated with perceived program effectiveness (*β* = −.28, *P* < .01). Further analyses showed that program content (*β* = −.85, *P* < .01) had a significant predictive effect on program effectiveness while this relationship was not significant in program implementer (*β* = −.01, *P* > .05). This model explained 71% of the variance toward the prediction of program effectiveness.

## 4. Discussion 

The present findings revealed that the program participants generally rated their participation in the program positively. In line with the previous findings using various methods and collected from different sources [24–30], the majority of the participants reported that they were satisfied with the program content, had an enjoyable experience, and perceived the program as beneficial to develop personal and social competencies. The present study provided support for the hypothesis that perceptions of the program content and program implementers were positively associated with program effectiveness. Findings suggested that participants' needs and interests were satisfied as they would participate again or recommend similar programs to their peers in the future. Taken as a whole, evaluation findings in the extension phase are highly similar to those reported in the original phase. From a triangulation point of view, data collected from different sources based on different methods generally suggest that the program is well received by different stakeholders.

The second aim of the study was to examine the relative influence of two program implementation factors (i.e., perceived program attributes and program implementers) on program effectiveness. Results of the regression analyses indicated that program content but not program implementers had a significant predictive effect on program effectiveness outcome. In line with previous studies [8, 9, 37–39], clear objectives of the curriculum, provision of well-designed teaching activities, participants' active participation, and perception of a motivated learning environment were associated with program outcomes. The findings indicated the importance of a well-planned program and the success of eliciting participants' engagement for program effectiveness. 

Another purpose of the study was to examine whether the relationships between program implementation factors and program evaluation outcomes would vary by the students' grade levels. Consistent with the previous study [34], Secondary 1 students perceived the program more favorably as compared to their higher grade level counterparts (i.e., Secondary 2 and 3 students). This observation might be related to the characteristics of the students. Compared to Secondary 3 students, Secondary 1 students were new to the project, and they were more interested and motivated to learn and participate in the program activities. Also, senior students were likely to act critically and engage in rebellious behaviors during this period of stress. Nevertheless, the differences observed were not great, and further studies to examine the related phenomena are needed.

It is interesting to note the negative predictive effect of program implementers on perceived program effectiveness. Some might question whether this result was related to the program implementers' teaching background. It is noteworthy that all program implementers of the Tier 1 Program were all experienced teachers and frontline social workers who had at least 3 years of experience in working with youths and received relevant formalized training workshops for more than 20 hours. Second, previous study [34] showed that program participants generally perceived program implementers positively (e.g., using effective and interactive teaching methods and skills, eliciting participants' learning motivation in the learning process, displaying enthusiasm in teaching). One possible explanation of this unexpected result might be related to the unit of analysis of the data. In the current study, data were aggregated at the school-level and the school means for each scale were computed and used for analysis. Clearly, it is important to examine this issue again using individual data rather than aggregate data. In addition, it would be helpful to test the associations between various dimensions of program implementation and program outcomes using advanced statistical technique. To increase the precision of measuring the effects of different program implementation factors on each level (e.g., students, classroom, and schools), future research should use multilevel statistical modeling to analyze the nested data (i.e., students nested within classrooms/schools).

While the present study focused on the influence of two program implementation factors, it is possible that other facilitators (e.g., fidelity, adaptation, dosage, reach) will also influence program effectiveness. For example, high levels of fidelity and increased cultural relevance of the program have been associated with program outcomes [6, 40, 41]. Program evaluation researchers noted the need of developing a theoretical model that identifies how different implementation factors exert their influence on program outcomes and thus untangle their conjointly effects on program effectiveness outcomes. Future research should include other factors in order to depict a comprehensive picture about the complex process of effective program implementation.

Providing a positive developmental experience in early adolescence would promote individuals' different competencies and reduce negative outcomes [42]. Consistent with western literature, a positive youth development program appears to be a promising approach to promote individuals' personal, emotional, social, and spiritual competencies and to deter a range of problem and risky behavior among Hong Kong adolescents [43, 44]. Understanding the factors underlying the complex program implementation process is critical in achieving their intended outcomes. The findings in the present study underscore the impact of program content and program implementers on perceived program effectiveness.

There are several limitations of this study. First, the use of self-report measure from a single perspective is limited to give a full picture concerning subjective outcome evaluation. However, this approach is commonly used in program evaluation research [6, 7, 9, 12]. In addition, reliability of the scales is very promising. Therefore, we could argue that the findings in the study are reliable and valid. Another limitation is the cross-sectional nature of the data. Future research should collect data at several points in time and also include predictors from various contexts, such as school and community. In particular, in seeking to monitor the rate of change of the perceived program effectiveness over time, growth curve modeling could be used to examine whether the predictive effects of the program implementation components on the shape of growth and the variability in individual trajectories would vary by the number of waves. However, in doing this, we must collect anonymous personal identifiers from the students. Third, aggregated data with schools with units of analyses rather than individual data were used in this study. Theoretically speaking, it would be interesting to look at the differences in the findings based on these two methods. Finally, ordinary least square analyses were used. As structural equation modeling may give a better estimation of the suppressors in the predictors, such techniques could be considered in future studies.

Despite the aforementioned limitations, the current study contributes to the positive youth development literature. It sheds light on what program components are associated with perceived program effectiveness. Shek et al. [45] argued that more research work is needed on subjective outcome evaluation, especially in social work education. To promote the dissemination of efficacious programs, it is important to consider characteristics of the participants. As supported by Catalano et al. [22], “if we are to discern why these (positive youth development) programs are effective, it is clear that it will be important in the future for programs to define and assess implementation methods and change strategies, and that they also evaluate the impact on youth development constructs…. and how these effects varied by subgroups” (page S94). The findings of the study attempt to address this gap in the program evaluation research. It provides insights to practitioners when designing and implementing effective positive youth development programs for Chinese adolescents. Most importantly, in conjunction with the previous findings, the present findings show that the influence of program attributes and program implementers on program effectiveness is relatively stable in different cohorts of students in Hong Kong [46–50].

## Figures and Tables

**Table 1:** Summary of the program participants' perceptions toward the program content.

	Respondents with positive responses (options 4–6)							
	S1		S2		S3		Overall	
	*n*	%	*n*	%	*n*	%	*N*	%
(1) The objectives of the curriculum are very clear	26,181	85.98	22,387	84.33	21,933	85.66	70,501	85.32
(2) The design of the curriculum is very good	25,224	82.88	21,351	80.49	21,176	82.72	67,751	82.03
(3) The activities were carefully planned	25,664	84.47	21,779	82.21	21,504	84.10	68,947	83.59
(4) The classroom atmosphere was very pleasant	24,977	82.29	21,568	81.56	21,410	83.81	67,955	82.55
(5) There was much peer interaction amongst the students	25,078	82.86	21,612	81.95	21,380	83.90	68,070	82.90
(6) I participated actively during lessons (including discussions, sharing, games, etc.)	25,110	82.60	21,246	80.26	21,048	82.36	67,404	81.74
(7) I was encouraged to do my best	24,085	79.24	20,425	77.08	20,358	79.63	64,868	78.65
(8) The learning experience I encountered enhanced my interest toward the lessons	24,168	79.70	20,425	77.24	20,398	79.96	64,991	78.97
(9) Overall speaking, I have very positive evaluation of the program	24,092	79.33	20,592	77.76	20,489	80.16	65,173	79.08
(10) On the whole, I like this curriculum very much	24,305	80.25	20,672	78.22	20,530	80.49	65,507	79.65

All items are on a 6-point Likert scale with 1: strongly disagree; 2: disagree; 3: slightly disagree; 4: slightly agree; 5: agree; 6: strongly agree. Only respondents with positive responses (options 4–6) are shown in the table. S1: Secondary 1 level; S2: Secondary 2 level; S3: Secondary 3 level.

**Table 2:** Summary of the program participants' perceptions toward the program implementers.

	Respondents with positive responses (options 4–6)							
	S1		S2		S3		Overall	
	*n*	%	*n*	%	*n*	%	*N*	%
(1) The instructor(s) had a good mastery of the curriculum	26,689	87.72	22,957	86.62	22,661	88.66	72,307	87.67
(2) The instructor(s) was well prepared for the lessons	27,119	89.15	23,263	87.75	22,822	89.29	73,204	88.73
(3) The instructor(s)' teaching skills were good	26,688	87.87	22,676	85.62	22,421	87.83	71,785	87.11
(4) The instructor(s) showed good professional attitudes	27,077	89.10	23,204	87.56	22,805	89.31	73,086	88.66
(5) The instructor(s) was very involved	27,283	89.76	23,400	88.38	23,007	90.17	73,690	89.44
(6) The instructor(s) encouraged students to participate in the activities	27,255	89.73	23,265	87.80	22,842	89.46	73,362	89.00
(7) The instructor(s) cared for the students	26,602	87.60	22,726	85.79	22,384	87.66	71,712	87.02
(8) The instructor(s) was ready to offer help to students when needed	27,101	89.24	23,235	87.74	22,882	89.61	73,218	88.86
(9) The instructor(s) had much interaction with the students	26,127	85.99	22,429	84.68	22,205	86.99	70,761	85.89
(10) Overall speaking, I have very positive evaluation of the instructors	27,016	88.83	23,326	87.97	22,915	89.67	73,257	88.82

All items are on a 6-point Likert scale with 1: strongly disagree; 2: disagree; 3: slightly disagree; 4: slightly agree; 5: agree; 6: strongly agree. Only respondents with positive responses (options 4–6) are shown in the table. S1: Secondary 1 level; S2: Secondary 2 level; S3: Secondary 3 level.

**Table 3:** Summary of the program participants' perception toward the program effectiveness.

The extent to which the Tier 1 Program (i.e., the program in which all students have joined) has helped your students	Respondents with positive responses (options 3–5)							
	S1		S2		S3		Overall	
	*n*	%	*n*	%	*n*	%	*N*	%
(1) It has strengthened my bonding with teachers, classmates, and my family	24,664	81.03	20,825	78.78	20,605	80.71	66,094	80.17
(2) It has strengthened my resilience in adverse conditions	25,200	82.87	21,329	80.69	20,963	82.13	67,492	81.90
(3) It has enhanced my social competence	25,833	85.00	21,681	82.15	21,390	83.86	68,904	83.67
(4) It has improved my ability in handling and expressing my emotions	25,569	84.14	21,567	81.69	21,260	83.37	68,396	83.07
(5) It has enhanced my cognitive competence	25,592	84.28	21,538	81.69	21,139	82.87	68,269	82.95
(6) My ability to resist harmful influences has been improved	26,187	86.22	22,190	84.03	21,647	84.88	70,024	85.04
(7) It has strengthened my ability to distinguish between the good and the bad	26,472	87.15	22,442	85.02	21,909	85.94	70,823	86.04
(8) It has increased my competence in making sensible and wise choices	26,289	86.54	22,137	83.86	21,685	85.04	70,111	85.15
(9) It has helped me to have life reflections	25,328	83.42	21,678	82.14	21,442	84.13	68,448	83.23
(10) It has reinforced my self-confidence.	25,057	82.50	20,920	79.25	20,552	80.64	66,529	80.80
(11) It has increased students' self-awareness	25,465	83.87	21,382	81.08	21,051	82.60	67,898	82.52
(12) It has helped students to face the future with a positive attitude	25,749	84.82	21,675	82.16	21,423	84.14	68,847	83.71
(13) It has helped students to cultivate compassion and care about others	25,591	84.29	21,775	82.56	21,420	84.01	68,786	83.62
(14) It has encouraged students to care about the community	24,984	82.28	21,128	80.10	20,830	81.70	66,942	81.36
(15) It has promoted students' sense of responsibility in serving the society	25,309	83.34	21,336	80.85	20,962	82.19	67,607	82.13
(16) It has enriched the overall development of the students	26,189	86.21	22,189	84.10	21,829	85.67	70,207	85.33

All items are on a 5-point Likert scale with 1: unhelpful; 2: not very helpful; 3: slightly helpful; 4: helpful; 5: very helpful. Only respondents with positive responses (options 3–5) are shown in the table. S1: Secondary 1 level; S2: Secondary 2 level; S3: Secondary 3 level.

**Table:** (a) If your friends have needs and conditions similar to yours, will you suggest him/her to join this course?

Respondents with positive responses (options 3-4)							
S1		S2		S3		Overall	
*n*	%	*n*	%	*n*	%	*N*
24,324	81.92	19,886	76.41	19,326	77.32	63,536	78.55

The item is on a 4-point Likert scale with 1: definitely will not suggest; 2: will not suggest; 3: will suggest; 4: definitely will suggest. Only respondents with positive responses (options 3-4) are shown in the table. S1: Secondary 1 level; S2: Secondary 2 level; S3: Secondary 3 level.

**Table:** (b) Will you participate in similar courses again in the future?

Respondents with positive responses (options 3-4)							
S1		S2		S3		Overall	
*n*	%	*n*	%	*n*	%	*N*
21,242	71.50	17,003	65.30	16,647	66.57	54,892	67.79

The item is on a 4-point Likert scale with 1: definitely will not teach; 2: will not teach; 3: will teach; 4: definitely will teach. Only respondents with positive responses (options 3-4) are shown in the table. S1: Secondary 1 level; S2: Secondary 2 level; S3: Secondary 3 level.

**Table:** (c) On the whole, are you satisfied with this course?

Respondents with positive responses (options 4–6)							
S1		S2		S3		Overall	
*n*	%	*n*	%	*n*	%	*N*
25,978	87.05	22,005	84.23	21,452	85.66	69,435	85.65

All items are on a 5-point Likert scale with 1: unhelpful; 2: not very helpful; 3: slightly helpful; 4: helpful; 5: very helpful. Only respondents with positive responses (options 3–5) are shown in the table. S1: Secondary 1 level; S2: Secondary 2 level; S3: Secondary 3 level.

**Table 5:** Mean, standard deviations, Cronbach's alphas, and mean of interitem correlations among the variables by grade.

	S1		S2		S3		Overall	
	*M*	*α*	*M*	*α*	*M*	*α*	*M*	*α*
	(SD)	(Mean^#^)	(SD)	(Mean^#^)	(SD)	(Mean^#^)	(SD)	(Mean^#^)
Program content (10 items)	4.37** (.29)	.98 (.84)	4.26** (.31)	.99 (.89)	4.33 (.32)	.99 (.90)	4.32 (.31)	.98 (.87)
Program implementers (10 items)	4.68** (.30)	.99 (.89)	4.55** (.31)	1.00 (.95)	4.61 (.31)	1.00 (.95)	4.61 (.31)	.99 (.93)
Program effectiveness (16 items)	3.50** (.25)	.99 (.91)	3.37** (.29)	1.00 (.95)	3.41** (.30)	1.00 (.96)	3.43 (.28)	1.00 (.94)
Total effectiveness (36 items)	4.07** (.25)	.99 (.76)	3.95** (.29)	1.00 (.85)	4.00* (.29)	.99 (.84)	4.01 (.28)	.99 (.82)

^#^Mean interitem correlations.

*P < .05; **P < .01; Bonferroni adjustment (*P* = .02). S1: Secondary 1 level; S2: Secondary 2 level; S3: Secondary 3 level.

**Table 6:** Correlation coefficients on the relationship between program components and program effectiveness.

Variable	S1	S2	S3	Overall
Program content (10 items)	.78**	.88**	.87**	.84**
Program implementers (10 items)	.73**	.78**	.76**	.76**

**P < .01. S1: Secondary 1 level; S2: Secondary 2 level; S3: Secondary 3 level.

**Table 7:** Multiple regression analyses predicting program effectiveness.

	Predictors			
	Program content	Program implementers	Model	
	**β** ^ a^	**β** ^ a^	*R*	*R* ^2^
S1	.62**	.18	.78	.61
S2	1.14**	−.28**	.89	.78
S3	.94**	−.08	.87	.76
Overall	.85**	−.01	.84	.71

^a^Standardized coefficients.

**P < .01. S1: Secondary 1 level; S2: Secondary 2 level; S3: Secondary 3 level.
